# Effects of gibberellic acid on Tifton 85 bermudagrass (*Cynodon* spp.) in constructed wetland systems

**DOI:** 10.1371/journal.pone.0206378

**Published:** 2018-10-26

**Authors:** Edcássio Dias Araújo, Alisson Carraro Borges, Neriamara Martins Dias, Dimas Mendes Ribeiro

**Affiliations:** 1 Department of Agricultural Engineering, Federal University of Viçosa, Viçosa, Minas Gerais, Brazil; 2 Department of Agricultural and Biological Engineering, University of Illinois at Urbana-Champaign, Urbana, Illinois, United States of America; 3 Department of Plant Biology, Federal University of Viçosa, Viçosa, Minas Gerais, Brazil; Estacion Experimental del Zaidin, SPAIN

## Abstract

This study aimed to evaluate 1) the influence of gibberellic acid (GA_3_) in the development of Tifton 85 bermudagrass grown in constructed wetland systems (CWs) and 2) the plant's capacity to remove nutrients and sodium from synthetic municipal wastewater (SMW). The experiment was carried out in Viçosa, Minas Gerais, Brazil, and consisted of foliar applications of GA_3_ set in randomized blocks design, with four replicates and 6 treatments as following: NC (control with plants); 0 μM GA_3_; N1: 5 μM GA_3_; N2: 25 μM GA_3_; N3: 50 and N4: 100 μM GA_3_ per CWs, NC* (control with no plants): 0 μM GA_3_. The study was conducted over two crop cycles in the spring 2016. The parameters used to evaluate the performance of the Tifton 85 bermudagrass were its plant height, productivity, chlorophyll measurement, number of internodes, nutrients and Na removals. Chemical analyses of the effluents were conducted. In response to the application of GA_3_, the increase in height of Tifton 85 bermudagrass in the first crop cycle was higher than the increase in height in the second crop cycle. The decrease in plant growth in response to GA_3_ in the second crop cycle may be linked to the age of the plant tissue and climatic conditions. The greater growth of the plants cultivated in the CWs allows a more efficient removal of pollutants, using simple management and low cost. The results suggest that applying 50 μM of GA_3_ to the development of Tifton 85 bermudagrass provides higher dry matter yield and removal of nitrogen, phosphorus, and sodium for the first crop cycle in CWs. However, in the second crop cycle, the application of GA_3_ had no effect on dry matter production and nutrient removal by Tifton 85 bermudagrass in CWs.

## Introduction

The disposal of wastewater into rivers, lakes and other water bodies leads to a reduction in quality of water resources, which are of prime importance to human and aquatic life. The discharge of wastewater contains high levels of organic nitrogen and phosphorus compounds that enable growth of algae and aquatic plants, which are responsible for eutrophication of water bodies.

Constructed wetland systems (CWs) are treatment systems planned and designed to use natural processes involving wetland vegetation, soils, and their associated microbial assemblages to improve water quality [1]. In CWs—under appropriate environmental conditions—physical, chemical and biochemical processes occur to treat sanitary sewage, industrial effluents and many types of wastewater [2]. Many countries have adopted these systems [3] because they require low-cost investments combined with their efficiency and feasibility [4].

The efficiency of sewage treatment using CWs is obtained through the hydraulic interaction of the substrate, plants, microorganisms and solar radiation. Pollutant removal takes place in a support media, which is responsible for the filtration and the formation of the biofilm next to the roots of the plants. The microorganisms adhere to the biofilm, degrade carbonaceous and nitrogenous matter, and the plants absorb the nutrients and heavy metals, thus promoting phytoremediation [5].

CWs have a high removal efficiency and appropriate effluent quality for final disposal, especially regarding organic pollutants (biochemical oxygen demand, chemical oxygen demand (COD), volatile suspended solids, and oils and greases). However, their efficiency of nitrogen (N) and phosphorus (P) removal is relatively low [3].

The selection of wetland plants is crucial to the success of the wastewater treatment because the plants uptake macro and micronutrients from the CWs for their development. The macronutrients N and P are essential for plant growth and development. Nitrogen, besides constituting proteins, is required to produce the molecule chlorophyll. P is important for the energy reserve and structural integrity of tissues.

Reed (*Phragmites australis*), cattail (*Typha spp*.), lakeshore bulrush (*Schoenoplectus lacustris*) and *Canna indica* are some of the most common wetland species studied in treatment wetlands [6]. Ornamental species [7] or species of zootechnical interest such as elephant grass (*Pennisetum purpureum*) and Tifton 85 bermudagrass (*Cynodon* spp.) [8] have been used in previous studies for economic return. Tifton 85 bermudagrass is a cross between bermudagrass (*Cynodon dactylon*) and a close tropical relative called stargrass (*Cynodon nlemfuensis*) with high zootechnical and economic potential due to its good palatability, digestibility, and expressive crude protein concentrations. In addition, it presents an attractive commercial value for the hay production, reaching higher values in regions with little forage availability.

Tifton 85 bermudagrass has interesting characteristics for wastewater treatment in CWs, as it is a perennial crop that presents a high growth rate, which in turn allows frequent cuts making it possible to extract large amounts of nutrients from the system [9].

One of the main mechanisms responsible for the removal of N in CWs is the uptake by plants when frequent cuts are performed [10]. Therefore, it is important to create favorable conditions for plants, such as adequate climatic conditions, plant density, cutting height, and the use of cultural practices that stimulate growth and development of plants to potentiate the extraction of nutrients in the CWs, thus generating a better-quality effluent.

In this context, since the nutrient removal is due to the fast plant growth and dry matter production, it is necessary to look for alternatives that potentiate the growth rate of the plants.

The growth rate of plants can be increased with the use of growth-regulating substances [11]. Gibberellic acid (GA_3_) is one of the classes of growth phytohormones that influence plant growth, increase elongation and cell division, which is evidenced by increased length and number of cells [12]. However, the effect of GA_3_ is influenced by several factors, such as environmental conditions, GA_3_ concentration, number of applications, and the season of application and species or cultivar [11].

One of the roles played by plants in CWs is nutrient removal. Nutrient uptake affects plant growth rate. However, there are no references in the scientific literature regarding the effect of GA_3_ on plants grown in CWs. Therefore, it is necessary to investigate the effects GA_3_ has on productive characteristics of Tifton 85 bermudagrass, and pollutant removal efficiency in CWs.

This study aimed to evaluate 1) the influence of GA_3_ in the development of Tifton 85 bermudagrass grown in CWs; and 2) the plant's capacity to remove nutrients and sodium (Na) from synthetic municipal wastewater (SMW).

## Materials and methods

### Characterization of the experimental area

The experiment was carried out in a greenhouse located at the experimental area of the water resources reference center belonging to the Department of Agricultural Engineering at the Federal University of Viçosa, (altitude: 650 m; latitude 20°45'14'' S, longitude 42°52'53'' W) Viçosa, Minas Gerais state, Brazil. The climate, according to the Köppen climate classification, is Cwa (humid subtropical climate with dry winter and hot summer).

**Fig 1:**
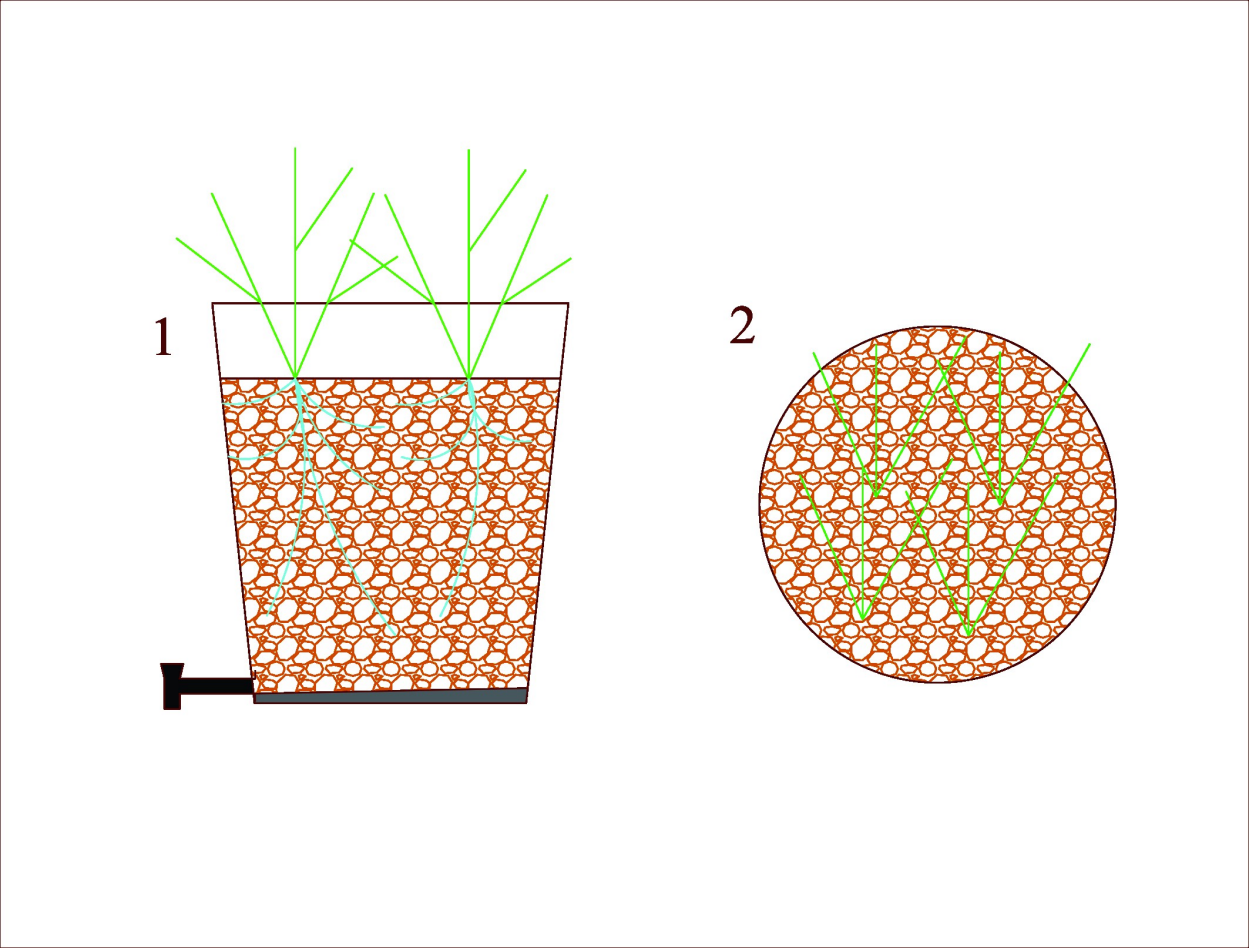
Scheme of the experimental system, 1—cross section and 2—upper view of the system.

The experimental system consisted of a set of 10 L polyethylene containers (reactors) with 26.50 cm height, 23.58 cm diameter, with a surface area of 0.0437 m^2^, cultivated with Tifton 85 bermudagrass (*Cynodon dactylon* Pers. x *Cynodon nlemfuensis* Vanderyst, Viçosa, Brazil), which were then submitted to SMW applications (Fig 1).

The CWs were filled up to 21.2 cm height with gneissic gravel 0 (D60 = 7 mm, coefficient of uniformity D60/D10 = 1.3, and initial void volume of 45.8%) and a free edge of 5.3 cm. Prior to filling the CWs, the rocks were immersed in a bleach solution and rinsed with running water.

### Experimental design and treatments

The different treatments and their respective doses of GA_3_ are listed in Table 1.

**Table 1:** Treatment with the application of GA_3_.

Treatments	Gibberellic acid doses (μM)
Level Control with grass (NC)	0
Level 1 (N1)	5
Level 2 (N2)	25
Level 3 (N3)	50
Level 4 (N4)	100
Level Control without grass (NC*) ^1^	0

^1^ Control with no Tifton 85 bermudagrass to assess the effluent.

A randomized complete block design was used, with four replications and five treatments with Tifton 85 bermudagrass exposed to different doses of GA_3,_ and one treatment control with no plants to assess the effluent, totaling 24 experimental plots.

### Synthetic municipal wastewater and operation of constructed wetland systems

The SMW was used in the experiment to guarantee greater control of chemical and organic compounds, and for sanitary purposes, since the operation of CWs and handling of SMW was done manually.

The SMW used in this study was modified from Nopens [13]; the concentrations of COD, N and P were consistent with the ranges recommended by Von Sperling for municipal wastewater [14], and the concentration of potassium (K) was around the values described by Santos [15]. The ingredients were diluted in drinking water. The compounds used to prepare SMW are shown in Table 2.

**Table 2:** Compounds and their theoretical concentrations to prepare 1 liter of synthetic municipal wastewater. **Source: Adapted from Nopens** [13].

Salts	Amount^1^	COD	N	P	K
	mg L^-1^				
Urea	92	23	43	0	0
Monoammonium phosphate	13	0	1	3	0
Sodium acetate	132	79	0	0	0
Peptone	17	17	1	0	0
MgSO_4_	20	0	0	0	0
KH_2_PO_4_	23	0	0	5	7
KCl	25	0	0	0	13
**Ingredients**					
Starch	122	122	0	0	0
Milk powder	116	116	7	1	0
Baking powder	52	52	6	0	0
Soybean oil	29	29	0	0	0
**Total**		438	58	9	20

^1^ Mass amount of ingredients, in mg, to make 1L of SMW.

Municipal wastewater is characterized by a high concentration of salts, especially K and Na. The concentrations of K and Na in the influent of the present study are similar to those found at the municipal wastewater treatment plant of Janaúba, Minas Gerais [15]. The concentration of Na in raw sewage ranges between 30 to 50 mg L^-1^ [16].

The CWs worked as a batch system, being fed from the top. Influent SMW was applied until the reactors were filled up to the point where the upper layer of gravel was reached. Then the volume of input of each CWs was recorded. SMW remained in reactors during a cycle time, then the SMW was collected from the bottom to characterize a vertical flow.

SMW was changed every 7 days (cycle time) during the acclimatization period of the experiment, and every 3.5 days after that. The cycle time in batch systems is analogous to the hydraulic residence time (HRT) in continuous systems.

The salts and the other ingredients were diluted into drinking water to prepare SMW; weekly evaluations of the parameters of the influent were conducted, totaling 11 analyses. The averages and standard deviations of the actual SMW composition in the present study are shown in Table 3.

**Table 3:** Parameters of the synthetic municipal wastewater influent.

Parameters	Units	Average	Standard Deviation
pH	-	6.7	0.4
EC	μS cm^-1^	336.6	33.5
TDS	mg L^-1^	224.7	24.9
COD	mg L^-1^	478.0	75.0
TN	mg L^-1^	56.0	3.6
TP	mg L^-1^	9.3	1.6
K	mg L^-1^	21.5	2.0
Na	mg L^-1^	24.9	3.1

Electrical conductivity (EC); total dissolved solids (TDS); chemical oxygen demand (COD); total nitrogen (TN); total phosphorus (TP), potassium (K) and sodium (Na).

After the acclimatization period, the differentiation of treatments with HRT of 3.5 d began and the average rates of SMW application were calculated (Table 4).

**Table 4:** Average rates of the synthetic municipal wastewater applications.

Parameter	^1^AR (g m^-2^ d^-1^)
TDS	4.10
COD	8.80
TN	1.02
TP	0.20
K	0.40
Na	0.50

^1^AR–application rate; total dissolved solids (TDS); chemical oxygen demand (COD); total nitrogen (TN); total phosphorus (TP), potassium (K) and sodium (Na).

### Experiment development and application of gibberellic acid (GA_3_)

Tifton 85 bermudagrass sprigs, formed by a segment of stolon with two buds with leaves and roots removed, were collected and standardized at 0.10 m. Then four sprigs were planted in each reactor.

The stabilization period lasted 48 days. At the end of this period, the plants were cut at a standard height of 0.10 m above the level of the support media. 0.1% (v/v) A spreader was added to the different doses of GA_3_ (2 drops of spreader in 25 ml GA_3_ solution) to facilitate the contact and absorption of the phytohormone by plants. The solutions were applied on the day of the standardization cut and at the end of the first cycle.

The first and second cycles lasted 18 and 23 days respectively and were carried out in the spring 2016 (southern hemisphere). The end of each cycle was pinpointed by when the Tifton 85 bermudagrass started lodging. Both cycles received two applications of the different doses of GA_3_; the second application was seven days after the plants being cut. The experiment was carried out for 89 days.

**Fig 2:**
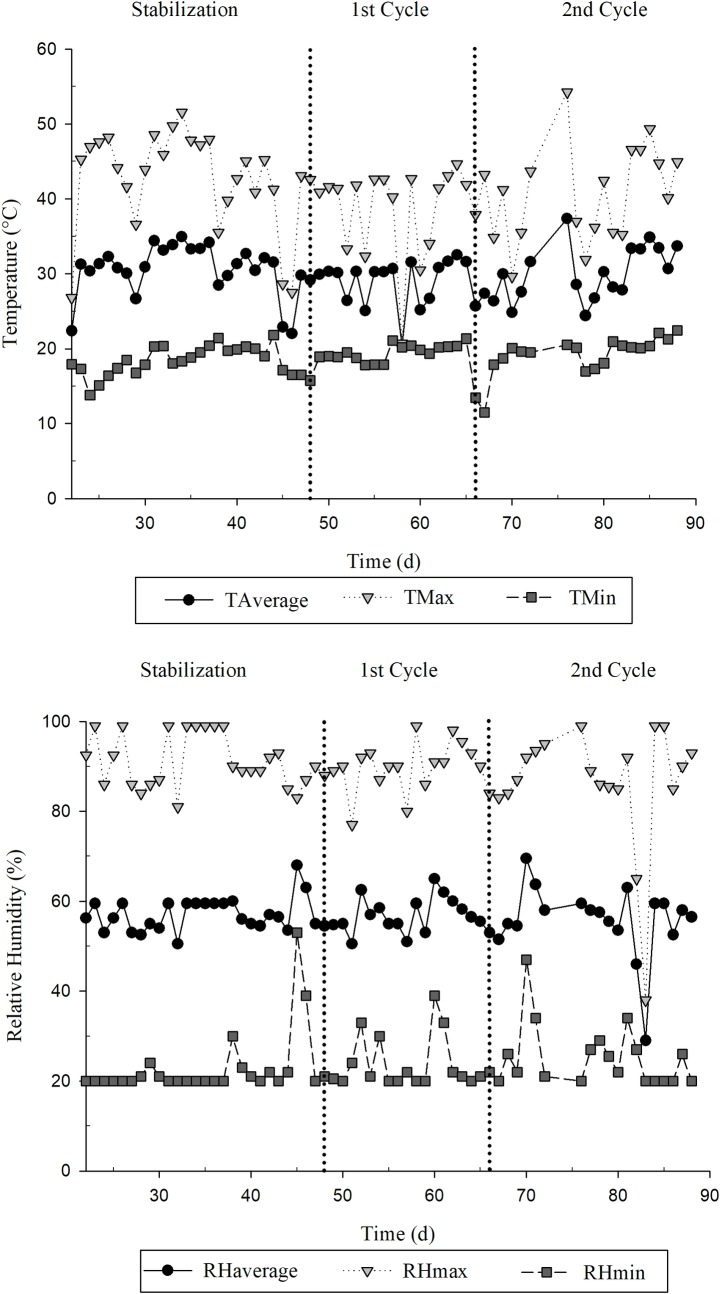
Temperature and relative humidity within the greenhouse.

The weather conditions (Fig 2) were measured with two thermohygrometers installed in the center of the greenhouse. Maximum and minimum and average temperatures were recorded to monitor the weather changes in all the stages of the experiment.

The weather conditions inside the greenhouse were not controlled, so in the first and second crop cycles, the average temperatures were 27.9 and 30.2° C, and the relative humidity was 58% and 56%, respectively.

### Experimental evaluations

#### Yield and vegetative analyses

The parameters analyzed in Tifton 85 bermudagrass and their methodologies are shown in Table 5. The forage yield was evaluated in two cycles when were performed cuts to 0.10 m at the level of the support media. The aerial parts of the material were collected and taken into a drying oven with forced ventilation at 65°C for 72 hours. At the end of the cycles, two grass stems per reactor were evaluated for each of the following parameters: plant height and number of internodes.

**Table 5:** Agronomic parameters analyzed and methods.

Parameters	Method
Height of plants	Measurement Tape
Dry Matter	Oven with forced ventilation
Chlorophyll	SPAD-502
Number of internodes	Counting

An estimate of chlorophyll content in leaves was determined at the end of each cycle using a chlorophyll meter. First, the chlorophyll meter was calibrated according to the specifications of the equipment, then SPAD readings were measured on one leaf per stem, on two distinct stems per reactor, on the middle third of the stem and the leaf blade.

#### Nutrients and sodium absorption by the Tifton 85 bermudagrass

The analyses of TN in the aerial part of the plants were quantified by the Kjeldahl semi-micro method with the addition of salicylic acid, adapted from Kiehl [17]. Absortion of K, TP and Na by plants followed the methods suggested by Silva [18].

Based on the analyses results, the nutrients/Na accumulation by Tifton 85 bermudagrass was calculated using Eq 1 [19].

$$A_{TN,K,TP\;or\;Na}=CNutri\times DMY$$ (1)

where, A_TN, K, TP or Na_—nutrient or Na accumulation (g), CNutri—content of nutrients or Na in dry matter of the aerial part of Tifton 85 bermudagrass (g g^-1^); DMY—dry matter of the aerial part of Tifton 85 bermudagrass.

The nutrients/Na uptake by Tifton 85 bermudagrass was calculated using Eq 2 [19].

$$U_{TN,K,TP\;or\;Na}=\frac{A_{TN,K,TP\;or\;Na}}{Vol}$$ (2)

where, U_TN, K, TP or Na_—nutrient or Na uptake (g m^-3^), A_TN, K, TP or Na_−nutrient or Na accumulation (g), Vol—CWs volume (m^3^).

The nutrients/Na uptake rate by Tifton 85 bermudagrass was calculated using Eq 3 [19].

$${UR}_{TN,K,TP\;or\;Na}=\frac{U_{TN,K,TP\;or\;Na}}{Ct}$$ (3)

where, UR_TN, K, TP or Na_–nutrient or Na uptake rate (g m^-3^ d^-1^), *U_TN,K,TP or Na_* – nutrient or Na uptake (g m^-3^), Ct—cycle time (d).

#### Effluent evaluations

In the experiments, influents and effluents were evaluated weekly, i.e., every two cycle times (HRT of 3.5 d). Analyses of oxidation-reduction potential, pH, electrical conductivity, evapotranspiration, COD, total nitrogen, total phosphorus, K, Na and total dissolved solids were performed to understand the current reactions and the nutrients and Na removal efficiency in CWs. The parameters analyzed are presented in Table 6.

**Table 6:** Parameters analyzed while monitoring CWs and methods.

Parameters	Method
Oxidation-reduction potential	Electrochemical probe
pH	Electrochemical probe
EC	Electrochemical probe
Evapotranspiration	Volume Balance Method
Chemical oxygen demand	Closed Reflux, Colorimetric Method
Total nitrogen	Semi-micro Kjeldahl
Total phosphorus	Spectrophotometry
Potassium	Flame Photometry
Sodium	Flame Photometry
Total dissolved solids	Electrochemical probe

The Tifton 85 bermudagrass evapotranspiration (ET_c_) was determined in each plot and after each cut (0.10 m). ET_c_ was calculated as the difference between the volumetric inflow and outflow divided by the surface area of the plot for data collected during the exchange of SMW, Eq 4 [20].

$$ETc=\frac{Qin-Qout}{SA}$$ (4)

where, ET_c_ (mm d^−1^), Q_in_ is the plot volumetric inflow (mm^3^ d^−1^), Q_out_, is the measured plot volumetric outflow (mm^3^ d^−1^), and SA is the measured plot surface (mm^2^).

The analyses of COD, TP_,_ K, and Na were performed according to the *Standard Methods for the Examination Water and Wastewater* [21]. TN of effluents was quantified according to the Kjeldahl semi-micro process with the addition of salicylic acid.

To obtain the actual value of the mass reduction in each parameter evaluated, the effluent concentrations of each CWs were corrected by ET_c_ (initial volume applied minus the final collected volume) and were calculated using Eq 5 [19].

$$Ce=CE*\frac{Vf}{Vi}$$ (5)

where, C_e_—corrected effluent concentration (g m^-3^), CE—effluent concentration (g m^-3^), V_f_−final volume collected (L), V_i_−initial volume applied (L).

The removal efficiency of SMW pollutants was calculated using Eq 6 [22].

$$RE=(\frac{Co-Ce}{Co})*100$$ (6)

where, RE—removal efficiency (%), C_0_—influent concentration (g m^-3^); C_e_—corrected effluent concentration (g m^-3^).

### Statistical analysis

All numerical data were subjected to variance analysis [23]. When they were significant at the 5% level, it proceeded to regression analysis and it was adopted the model with the highest coefficient of determination (R^2^) and that expressed the behavior of the phenomenon. The regression coefficients were subjected to *t-test* at the 5% level of significance. When the regression model presented the level above 5%, the probability found for the model was used. When the regression model did not explain the phenomenon, the Dunnett’s test was used to compare the GA_3_ treatments with the control.

## Results and discussion

### Yield and vegetative development of Tifton 85 bermudagrass

In the first cycle, the parameters plant height, dry matter yield (DMY) and Tifton 85 bermudagrass SPAD index were significantly influenced by various doses of GA_3_ (Table 7). In the second cycle, plant height was the only significant effect for the levels tested. The productivity of Tifton 85 bermudagrass had no significant effect for the second cycle; the same was true for the SPAD index and number of internodes.

**Table 7:** Summary of the analysis of variance for the parameters accumulation of yield and vegetative development of the Tifton 85 bermudagrass in the first and second cropping cycles.

FV	GL	PH	DMY	SPAD	Nint.
		**1st Cycle**			
**Blocks**	**3**	27.82	78442.96	6.46	0.25
**Treatment**	**4**	219.31**	1244664.33**	70.48**	0.33^ns^
**Residue**	**12**	10.91	202104.57	10.14	0.27
**Average**		**49.21**	**3634.93**	**38.14**	**4.48**
**CV (%)**	** **	**6.71**	**12.37**	**8.35**	**11.54**
		**2nd Cycle**			
**Blocks**	**3**	45.60	33247.67	0.84	0.83
**Treatments**	**4**	61.65**	304,445.23^ns^	2.91^ns^	0.18^ns^
**Residue**	**12**	5.81	126623.44	4.12	0.46
**Average**		**44.54**	**4830.43**	**36.40**	**4.30**
**CV (%)**	** **	**5.41**	**7.37**	**5.58**	**15.74**

Where:

^ns^on-significant

** significant at 1% by F test. Plant height parameters (PH), dry matter yield of the aerial part of the plants (DMY), chlorophyll measurement (SPAD), number of internodes (Nint.) of the first and second crop cycles.

**Fig 3:**
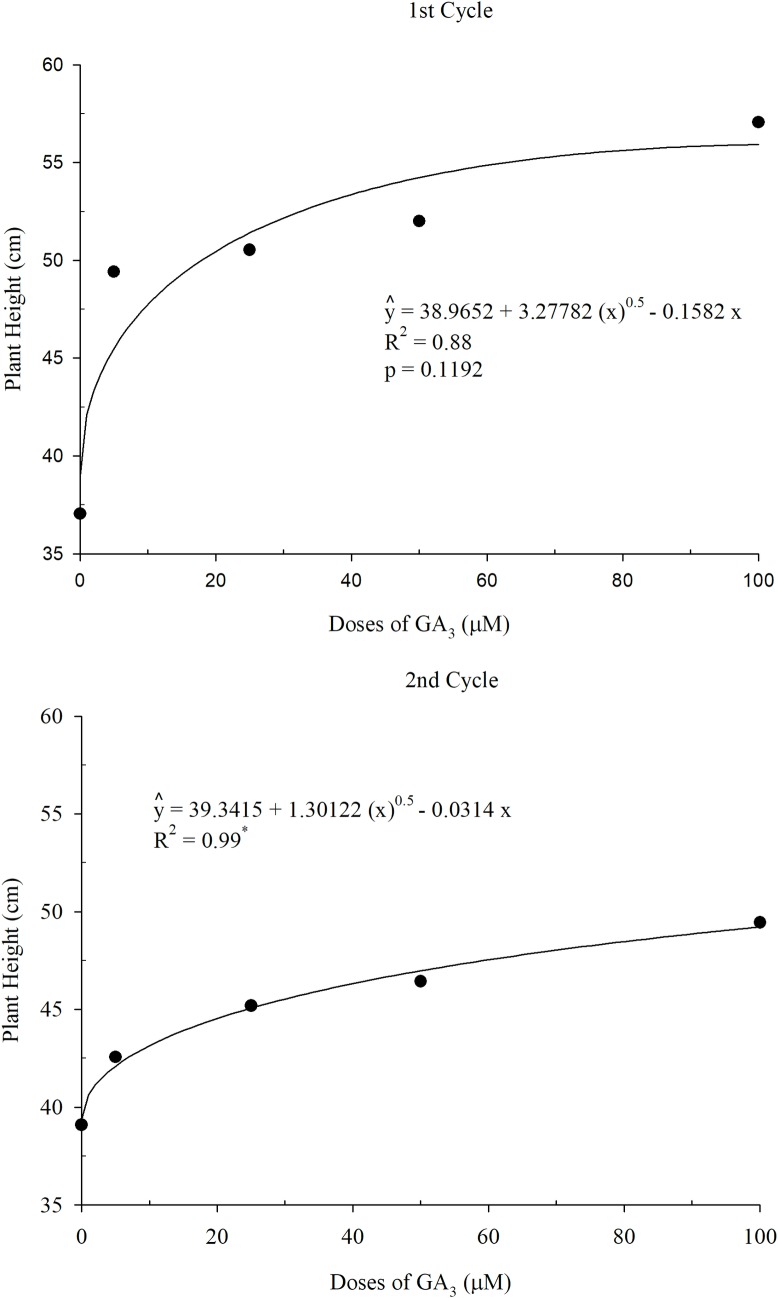
Plant height depending on the doses of GA_3_ (1st and 2nd cycles).

Fig 3 shows the values of Tifton 85 bermudagrass heights of two crop cycles, with a positive effect on growth according to the GA_3_ doses. A square root model regression was set for each cycle. For the 1st cycle, the model presented p = 0.1192 by t-test.

**Fig 4:**
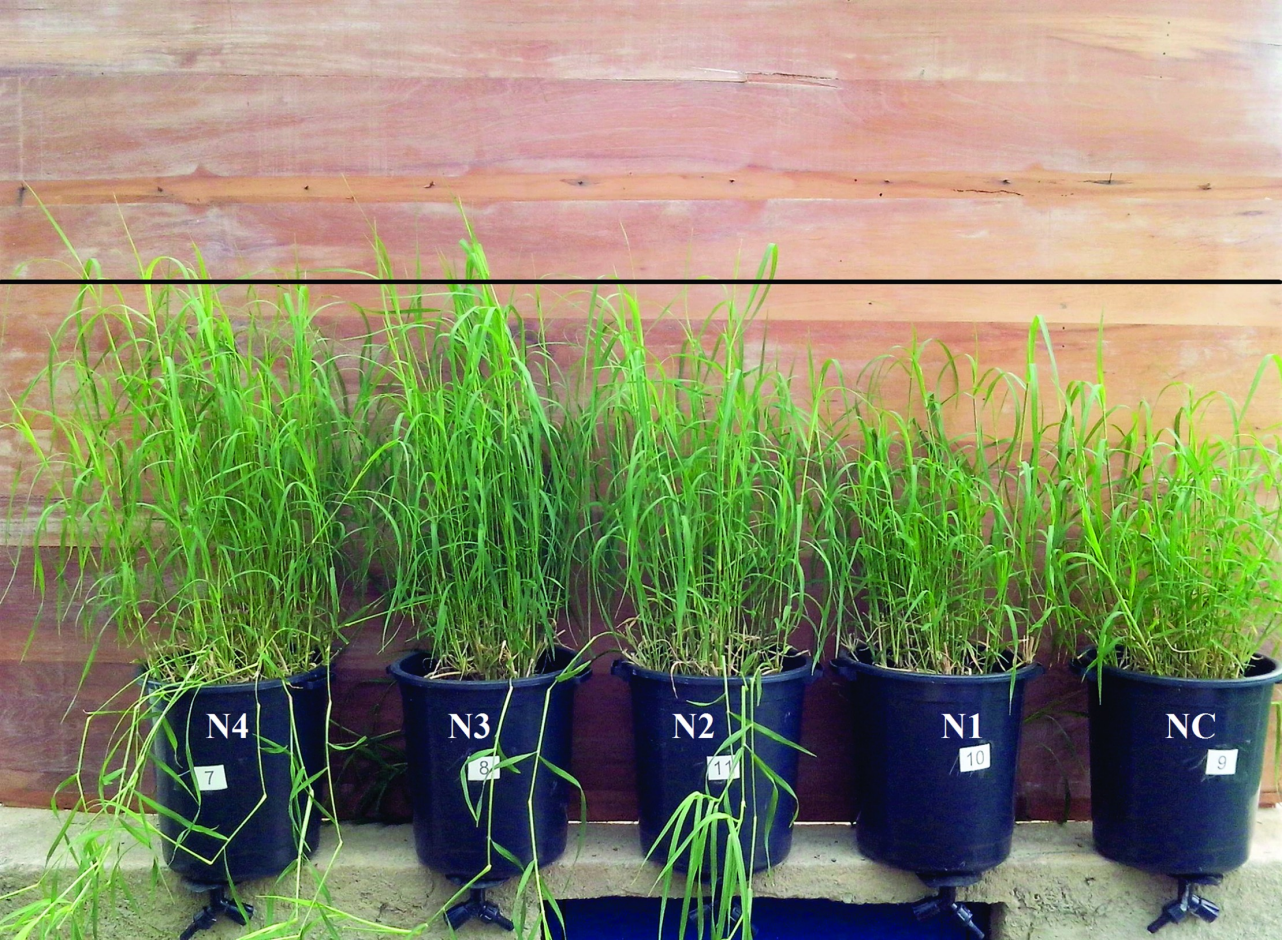
Tifton 85 bermudagrass treated with various doses of GA_3_ (2nd cycle). Treatments were NC = 0; N1 = 5; N2 = 25; N3 = 50 and N4 = 100 μM GA_3_.

One of the roles of GA_3_ is to promote elongation of the stem [12]. This effect was observed in this study for plant height of Tifton 85 bermudagrass (Figs 3 and 4). The increase in plant height is due to the lengthening of the stem internodes, as there was no difference in the number of internodes (Table 7). Comparing the best treatment for plant height (100 μM GA_3_) with the control, it is observed an increase of 35.10% and 20.90% in plant height for the first and second cycles, respectively.

In the two cycles observed, the control without the application of GA_3_ showed lower growth than the other treatments (Fig 3). The Tifton 85 bermudagrass is well suited for the conditions of CWs and may be able to develop aerenchyma in hypoxic conditions [24]. However, it is not considered an aquatic plant, and, in municipal wastewater treatment systems, this plant is grown in media with high concentrations of salts (K and Na). Thus, one can deduce that the grass is growing in abiotic stress conditions. When plants grow under abiotic stress conditions, they have their levels of phytohormones altered and, therefore respond with reduced growth [25].

When studying the effect of exogenous application of GA_3_ on dwarf rice, a significant increase in plant height was observed compared to a treatment without the phytohormone [26].

The increase in height of Tifton 85 bermudagrass in the first cycle was approximately 3.3 x^0.5^, being x the doses of GA_3_, though the increase in the same parameter for the second cycle was 1.3 x^05^. The reduced response to GA_3_ may be linked to the age of plants because the GA_3_ response in older tissue was less prominent. Gibberellins are produced in young tissues of the shoot system and developing seeds [27]. The effect of GA_3_ on plants is influenced by weather conditions [28]; possibly high temperatures in the second cycle negatively affected the induction of Tifton 85 bermudagrass growth.

**Fig 5:**
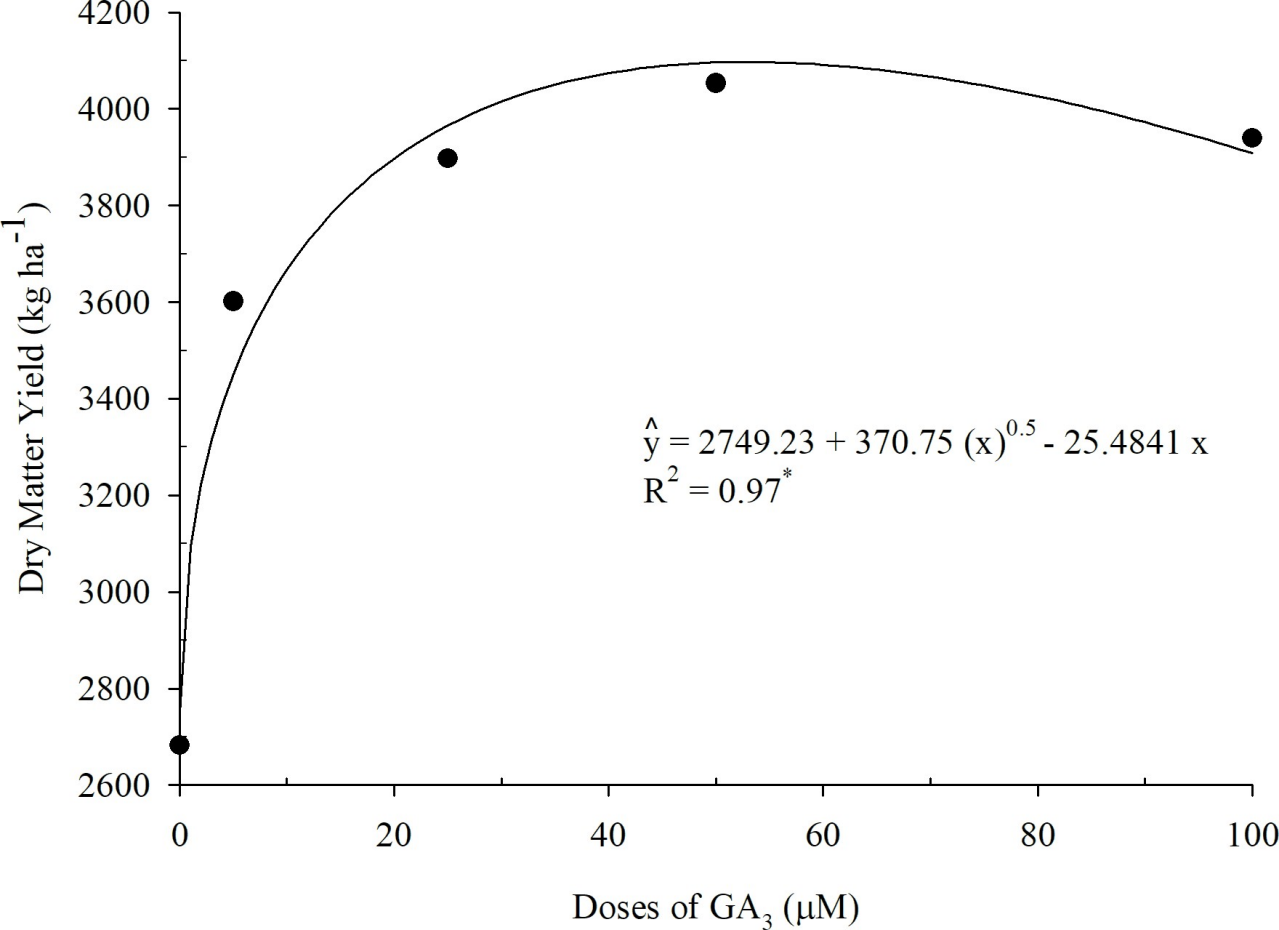
Tifton 85 bermudagrass dry matter yield in function of different doses of GA_3_ (1st cycle).

The DMY of aerial parts of Tifton 85 bermudagrass in the first cycle presented a square root behavior for the various doses of GA_3_ (Fig 5). The highest productivity found in the regression model was 4097.70 kg ha^-1^ depending on the optimal dose of 53 μM GA_3_. It was observed in the control treatment (0 μM GA_3_) 2682.60 kg ha^-1^ yield, i.e., 33.80% lower than the yield obtained with the treatment of 50 μM of GA_3_.

The increase of the DMY in aerial parts of Tifton 85 bermudagrass with the exogenous application of increasing doses of GA_3_ indicated that the phytohormone plays an important role in the absorption of water and nutrients and processing the accumulation of carbohydrates within plant tissues. The same was observed by Zang during foliar application of GA_3_ and evaluation of dry matter in blueberry leaves [29].

The GA_3_ stimulates cell division and cell elongation and the exogenous addition of this phytohormone leads to an increase in plant growth, improving the availability of endogenous GA_3_. This view is supported by Kaur [30] and Tuna [31], working with chickpea and corn, respectively. A*rabidopsis thaliana* plants treated with GA_3_ showed a higher shoot development compared to the control treatment [32].

Plants have an important role in the removal of nutrients in CWs, however, the removal capacity can be improved if crop conditions that allow rapid growth and dry matter production are created [33]. In view of this, the application of GA_3_ is a viable practice to improve the development of plants, as well as to create conditions to increase the removal of nutrients (pollutants).

**Fig 6:**
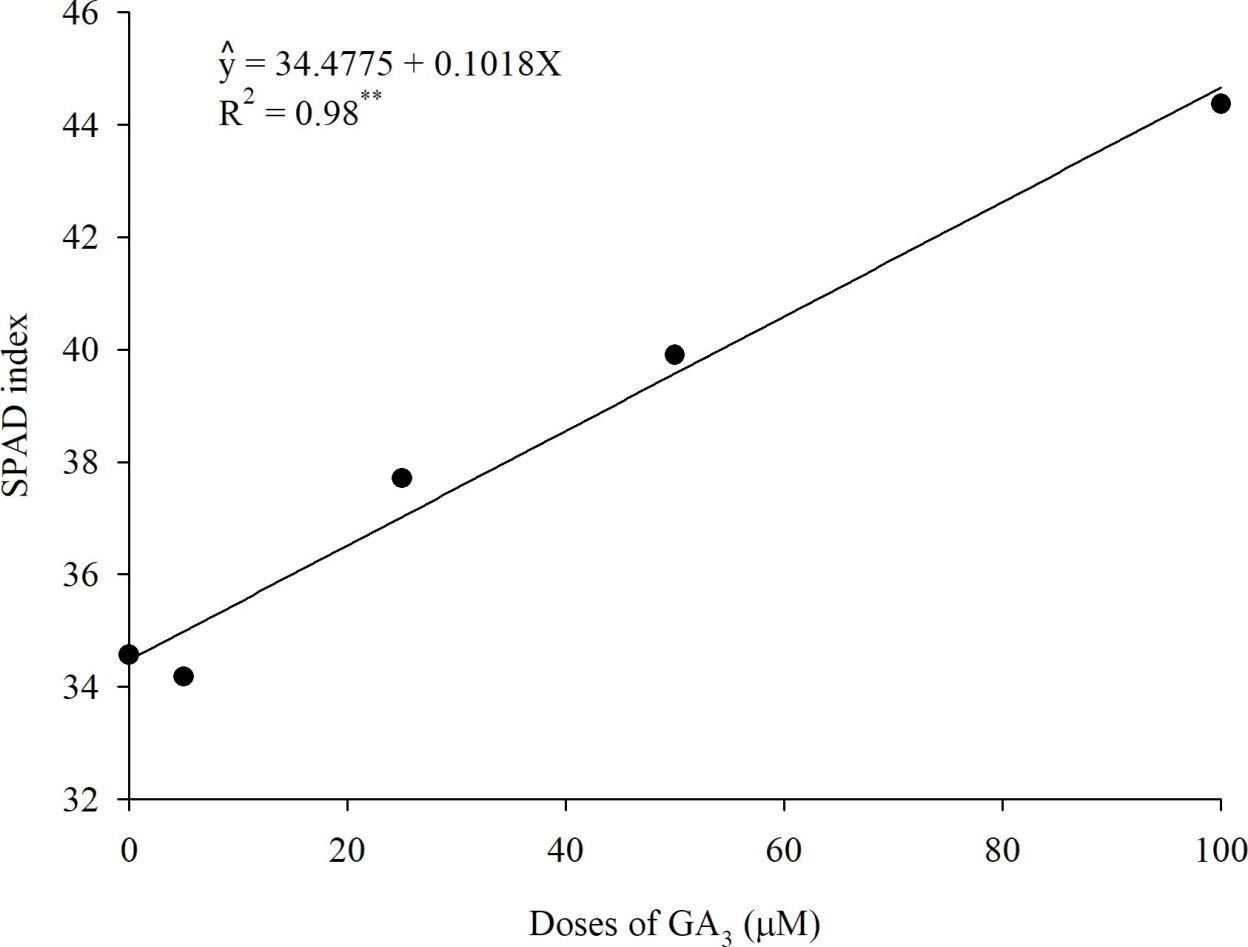
SPAD index as a function of different doses of GA_3_ (1st cycle).

As for the SPAD index evaluated in the first cycle (Fig 6), an increasing and linear behavior among the different doses of GA_3_ was observed_,_ indicating a lower chlorophyll content in the treatment without exogenous application of GA_3_.

Since the plants were grown in adverse conditions (flooded media and the presence of salts), this lower amount of chlorophyll in the control could be attributed to the formation of proteolytic enzymes such as chlorophyllase, an enzyme responsible for the degradation of chlorophyll [31], which may damage the photosynthetic system. The application of GA_3_ improved chlorophyll levels in plants grown in CWs.

The absence of effect of GA_3_ for DMY and SPAD index in the second cycle may be related to the period of application of the phytohormone. Some studies reported that GA_3_ applied during the spring season promotes a high forage growth and that response to the hormone is reduced in summer due to the high temperatures [28]. As can be seen in Fig 2, the maximum and average temperatures in the second crop cycle (near the summer season) were higher than the temperatures in the first cycle. Other factors which may have influenced the absence of effect of GA_3_ in the second cycle are the age of the plant, space restriction for root growth, and a lower GA_3_ response in older tissues.

### Absorption of nutrients and sodium by Tifton 85 bermudagrass

The parameters, total nitrogen and total phosphorus accumulation, and total nitrogen uptake rate by Tifton 85 bermudagrass had a significant effect under the different doses of GA_3_ compared to the treatment without the application of GA_3_ (Table 8). The same parameters were evaluated in the second cycle and were not significant at the 5% level. That absence of significance could be correlated with the absence of the GA_3_ effect for the productive parameters of the second cycle.

**Table 8:** Summary of the analysis of variance for the parameters of nutrients/Na accumulation and nutrients/Na uptake rate by Tifton 85 bermudagrass in the first and second cropping cycles.

FV	GL	A_TN_	A_K_	A_TP_	A_Na_	UR_TN_	UR_K_	UR_TP_	UR_Na_
		**1st Cycle**							
**Block**	**3**	0.004	0.002	0.0001	0.00	0.174	0.071	0.002	0.0001
**Treat.**	**4**	0.02**	0.02^ns^	0.0009*	0.00^ns^	0.99**	1.03^ns^	0.04^ns^	0.002^ns^
**Residue**	**12**	0.004	0.011	0.0003	0.00	0.182	0.508	0.013	0.002
**Average**		**0.51**	**0.36**	**0.08**	**0.02**	**3.55**	**2.55**	**0.58**	**0.15**
**CV(%)**		**11.85**	**28.32**	**19.79**	**1.89**	**12.03**	**27.93**	**19.83**	**25.71**
		**2nd Cycle**							
**Block**	**3**	0.002	0.002	0.00	0.0001	0.054	0.600	0.0002	0.0011
**Treat.**	**4**	0.001^ns^	0.005^ns^	0.0001^ns^	0.0001^ns^	0.04^ns^	0.15^ns^	0.003^ns^	0.003^ns^
**Residue**	**12**	0.002	0.003	0.00	0.00	0.059	0.104	0.001	0.0009
**Average**		**0.64**	**0.42**	**0.10**	**0.04**	**3.52**	**2.30**	**0.56**	**0.21**
**CV(%)**		**6.86**	**13.81**	**6.30**	**16.77**	**6.93**	**13.98**	**6.45**	**14.35**

Where:

^ns^ non-significant

* and **, significant at 5 and 1%, respectively, the F test. Total nitrogen accumulation (g) (A_TN_), potassium accumulation (A_K_), total phosphorus accumulation (A_TP_) and sodium accumulation (A_Na_). Total nitrogen uptake rate (g m^-3^ d^-1^) (UR_TN_), potassium uptake rate (UR_K_), total phosphorus uptake rate (UR_TP_) and sodium uptake rate (UR_Na_).

The absence of significant parameters evaluated in the second crop cycle may have occurred due to the room limitation within the reactors for root growth as a function of plant age. Also, the height of the support media was 21.20 cm, while Matos, cultivating Tifton 85 bermudagrass in CWs, reported that they reached a depth of 30 cm [34]. Another factor that may have influenced the absence of significant responses was GA_3_ lower response in older tissues since this hormone is produced in young tissues [27].

It is worth mentioning that the experiment aimed to verify the effect of the GA_3_ application in the same cutting stage of the control without application, but one of the suitable practices of forage cultivation is to establish the cut by height and not by the time of cultivation, so it would be possible to perform more cuts when applied GA_3_ which would result in greater removals of the pollutants from the CWs.

**Fig 7:**
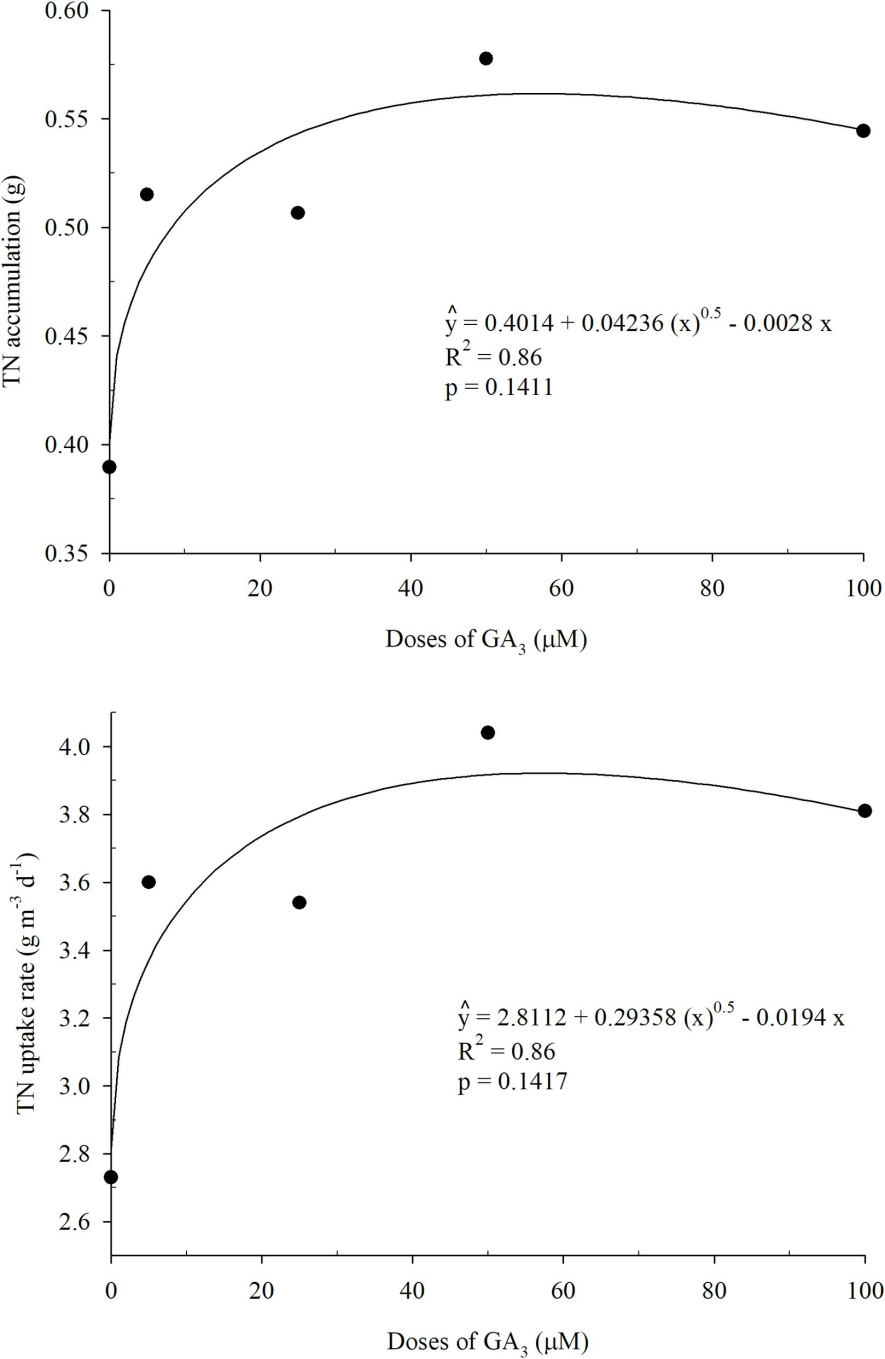
Total nitrogen accumulation and total nitrogen uptake rate by Tifton 85 bermudagrass as a function of the different doses of GA_3_ (1st cycle).

In Fig 7, the effects of the A_TN_ and UR_TN_ parameters were adjusted to the square root regression model with p = 0.1411 and p = 0.1417 respectively, making it possible to understand the relationship between GA_3_ doses and TN removal by plants.

For the generated model (Fig 7) the highest accumulation and TN uptake rate was 0.56 g and 3.92 g m^-3^ d^-1^ respectively, for the 57 μM dose of GA_3_.

In the first cycle, GA_3_ promoted an increase in the DMY of the aerial part of the plants. Associated to this effect, the plants accumulated larger amounts of N resulting in higher rates of removal of this nutrient (Fig 7). Mustard plants accumulated higher amounts of N in the shoots and seeds in treatments with the exogenous application of GA_3_ [35].

The application of GA_3_ in environmental conditions with a high concentration of atmospheric CO_2_ promoted a lower concentration of N in *Arabidopsis thaliana*. However, for environments with different concentrations of CO_2_, the DMY was higher for the GA_3_ treatments compared to the control. As a result, plants treated with GA_3_ had a higher accumulation of nitrogen [32].

The accumulation of TP in the aerial part of Tifton 85 bermudagrass is presented in Table 9. Because it did not present a significant regression model to explain the effect of the doses of GA_3_, the treatments with the control were compared by the Dunnett’s test, where only level 1 (5 μM GA_3_) differed from the control level without GA_3_ application.

**Table 9:** Total phosphorus accumulation of aerial part of Tifton 85 bermudagrass and Dunnett’s test for comparison of the treatments with doses of GA_3_ (1st Cycle).

Treatment	1st Cycle
	A_TP_
N1	0.10
N2	0.08^a^
N3	0.09^a^
N4	0.09^a^
NC	0.06^a^

The treatment averages followed by the letter ^(a)^ did not differ from the NC control by the Dunnett’s test at the 5% level of significance. A_TP_—accumulation of total phosphorus in aerial part of plants (g).

The treatment with exogenous application of GA_3_ in a maize crop under salinity conditions increased foliar TP [31]. The higher growth and productivity of Tifton 85 bermudagrass treated with GA_3_ compared to the control promoted a greater accumulation of TP for the N1 treatment. From the environmental point of view, it is an advantage since it showed that the removal of TP in CWs can be improved with an application of GA_3_.

### The efficiency of constructed wetland systems in the removal of pollutants from synthetic municipal wastewater

**Fig 8:**
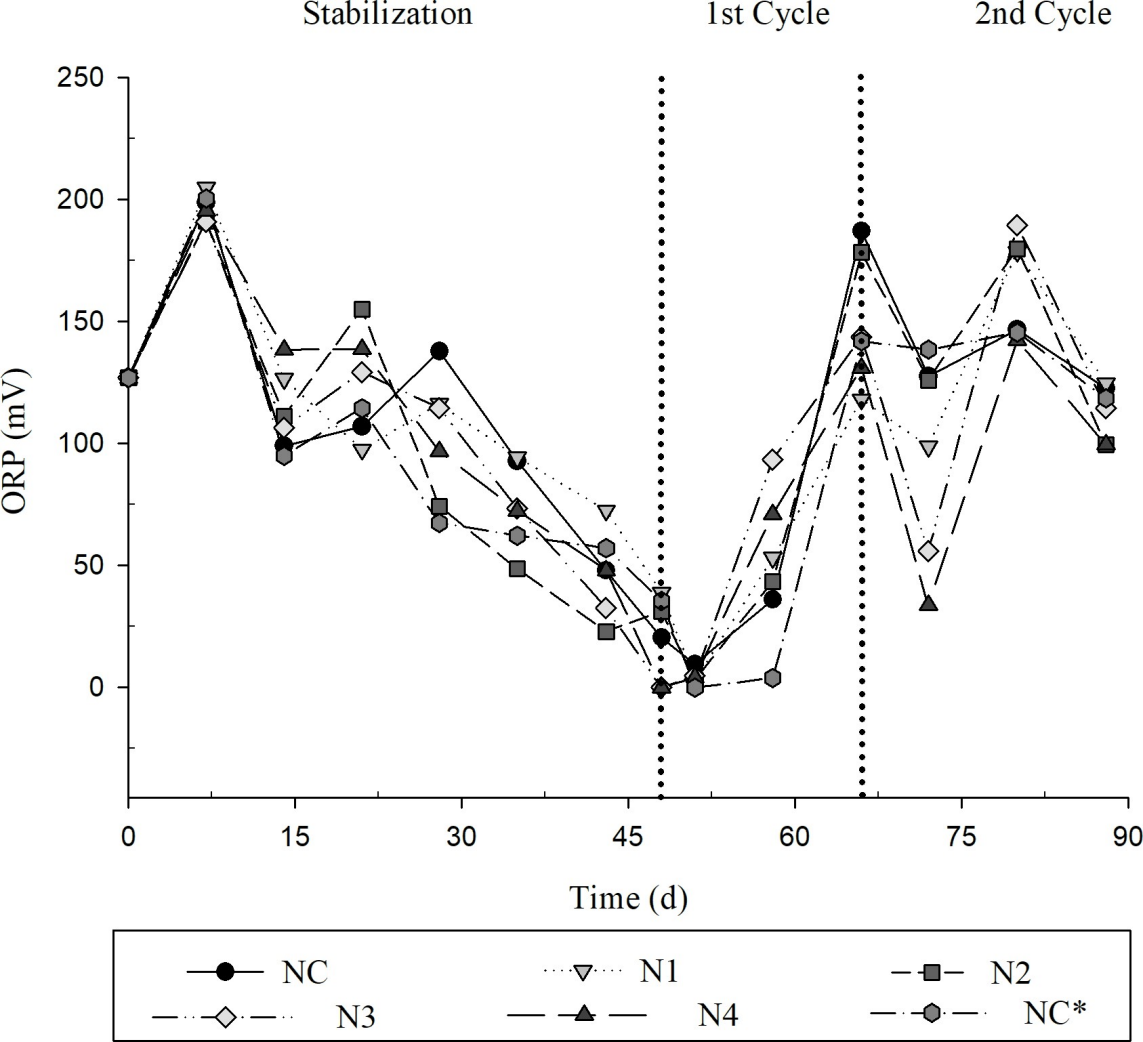
Oxidation-reduction Potential of sanitary effluent treated for the different treatments. NC = 0; N1 = 5; N2 = 25; N3 = 50 and N4 = 100 μM GA_3_; NC* control without the cultivation of Tifton 85 bermudagrass.

The influent had an average oxidation-reduction potential (ORP) of 127.2 mV. Only in three peaks (Fig 8), was it possible to observe ORP above the input value, indicating a small effect of the oxygenation promoted by the root system. This may be an indicator of the formation of aerenchyma by the roots of Tifton 85 bermudagrass. This formation in the plant tissue can be characterized as a mechanism of adaptation in flooded systems. Other studies have described and presented histological sections with the presence of aerenchyma in roots of Tifton 85 bermudagrass in flooded systems [24, 36].

ORP evaluated throughout the experimental period remained between 0–204.75 mV. A critical factor for nitrification would be an ORP above 500 mV [37].

In function of the ORP evaluated in the present study, it was possible to indicate that the conditions of the CWs were anoxic/facultative. A classification for the ORP of the medium, ORP above 400 mV has the predominance of O_2_ (oxidized medium); between 400–200 mV, the presence of O_2_ and nitrate (NO_3_^-^); and below 200 mV indicates low oxygen presence tending to anoxic/facultative conditions [38].

**Fig 9:**
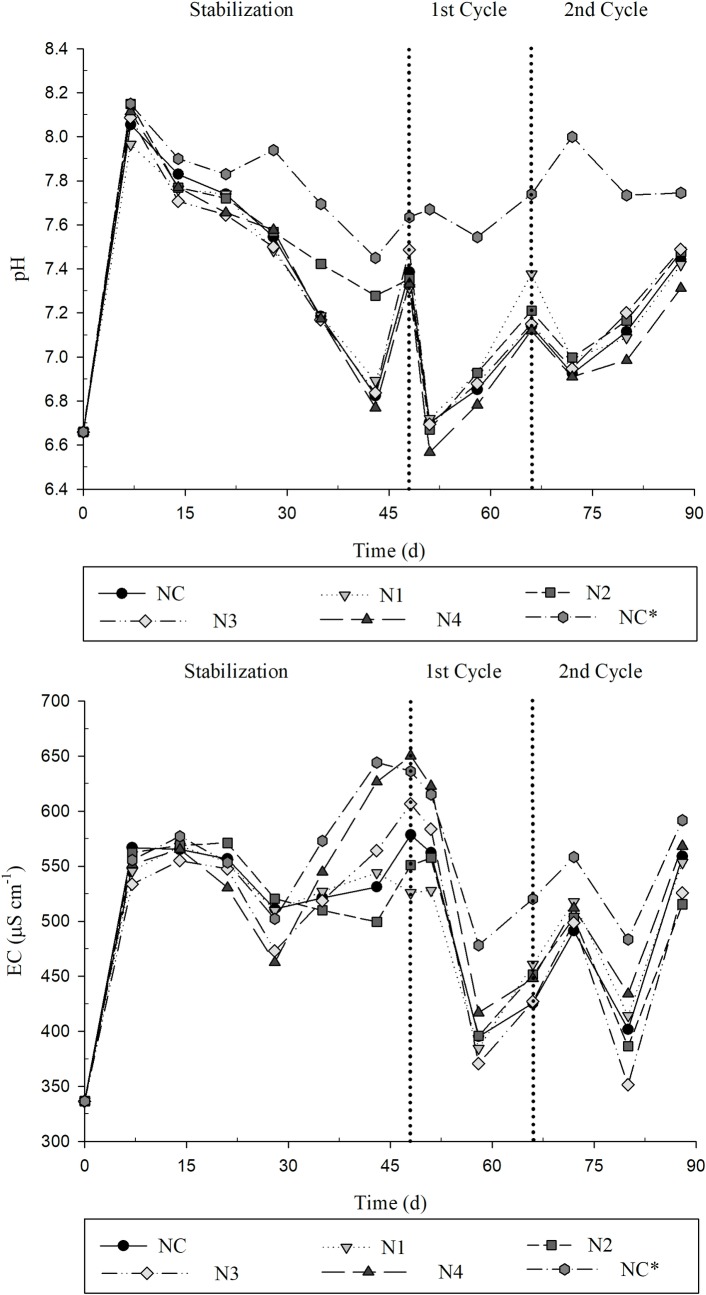
Potential of hydrogen and electrical conductivity of the effluents for the different treatments. NC = 0; N1 = 5; N2 = 25; N3 = 50 and N4 = 100 μM GA_3_; NC* extra control without the cultivation of Tifton 85 bermudagrass.

After collecting the effluents, the pH and EC were determined, and their values are presented in Fig 9. The pH of the inlet was on average 6.7 and the EC of 336.1 μS cm ^-1^. Except for the NC*, the treatments presented a similar behavior for the parameter pH and EC. In both cycles the pH and EC were higher for the NC*, this allows to understand the importance of the removal of the pollutants (nutrients and Na) and other salts by the plants.

The increase in pH in the different treatments may be related to the use of carbon dioxide by plants and algae [5].

The pH values ranged from 6.5 to 8.2 for treatments and control plots, what indicated that SMW (influent and effluent) presented ideal conditions for the survival of microorganisms and for the degradation of organic matter [39]. The optimum range for nutrient absorption is between 5.5 and 6.5, pH above 7.0 causes a great restriction in the availability of micronutrients and P [40].

**Fig 10:**
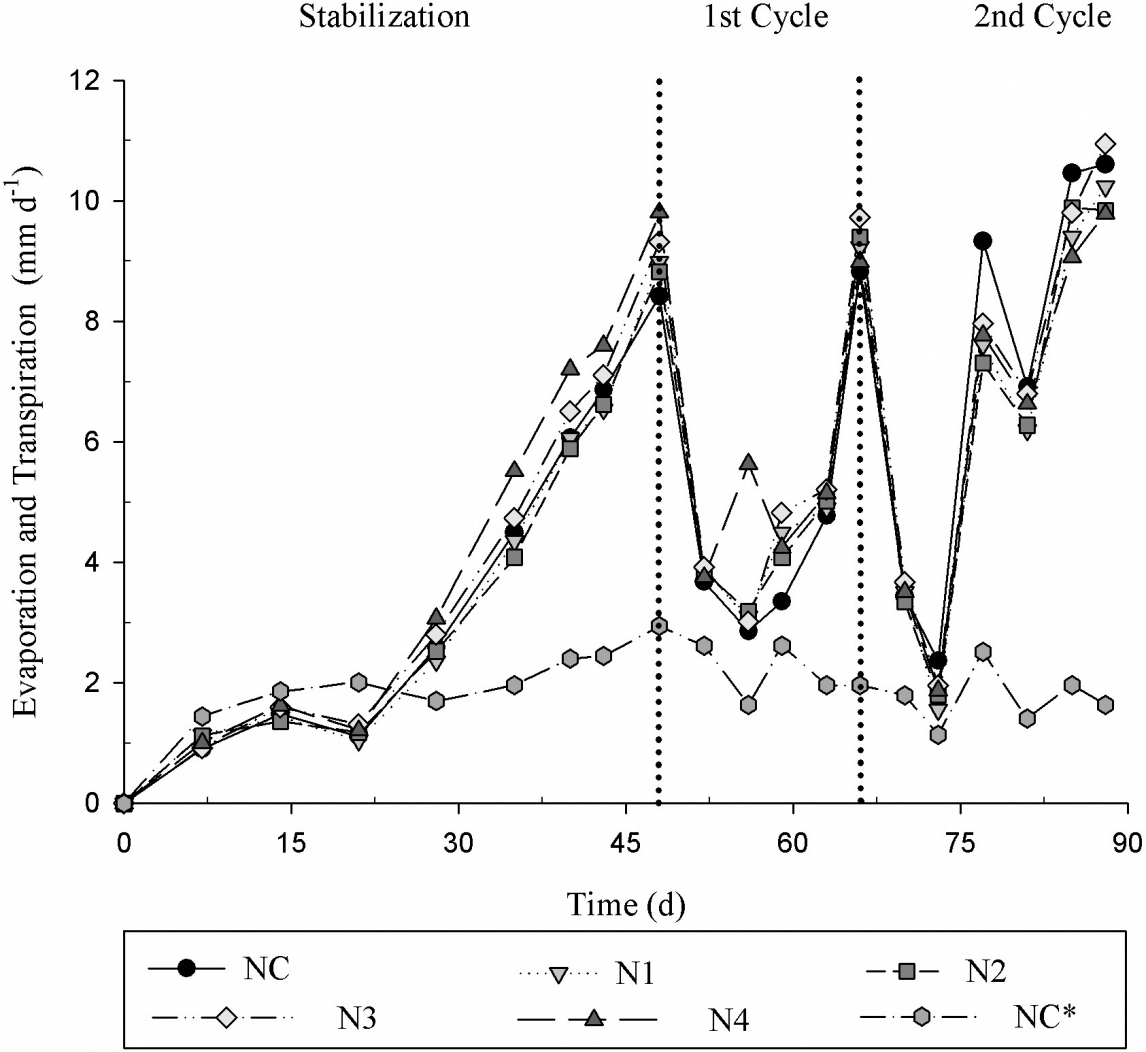
Evaporation and Transpiration for different treatments. NC = 0; N1 = 5; N2 = 25; N3 = 50 and N4 = 100 μM GA_3_; NC* control without the cultivation of Tifton 85 bermudagrass.

The daily average evaporation and ET_c_ was calculated through the determination of the volume of the input (influent) and the output (effluent) (Fig 10). Subsequently, the mass differences were calculated to express the removal of nutrients and Na. The system without Tifton 85 bermudagrass showed an average evaporation of 2.0 mm d^-1^ and the system with Tifton 85 bermudagrass grass showed an average ET_c_ of the treatments of 5.2 mm d^-1^.

It is possible to observe the importance of plant cultivation in CWs since they transform the SMW into water into the atmosphere through transpiration. Thus, in addition to removing the pollutants (nutrients), CWs with plants reduce the volume of sewage for the final disposal.

**Fig 11:**
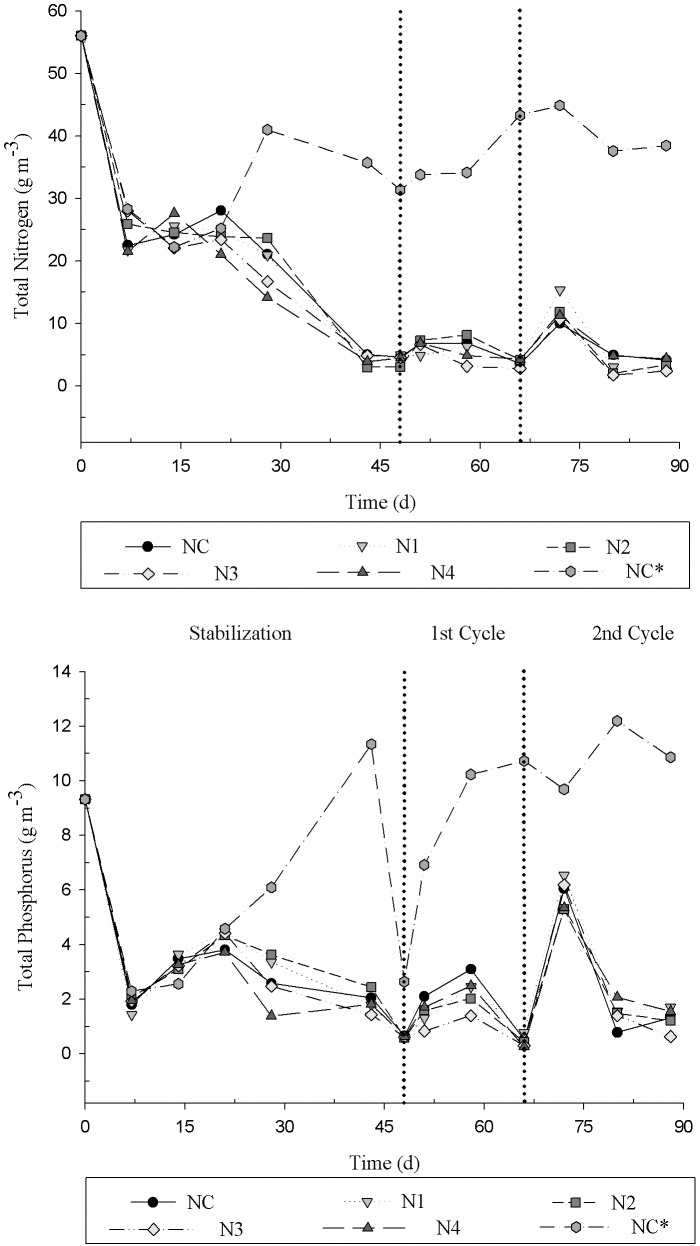
Total nitrogen and total phosphorus concentration, corrected as a function of ET_c_ of the effluent for the different treatments. NC = 0; N1 = 5; N2 = 25; N3 = 50 and N4 = 100 μM GA_3_; NC* control without the cultivation of Tifton 85 bermudagrass.

The behavior of TN and TP concentrations in the effluent is shown in Fig 11. Initially, the removal of TN and TP by plants compared to the control without plants was perceptible. The removal of these elements in CWs reduces the N and P disposal into rivers and lakes, these nutrients are essential for the growth and multiplication of algae that are responsible for the eutrophication of the water bodies. Therefore, plants play an important role in the uptake of N in the forms of NO_3_^-^ and ammonium (NH_4_^+^); the ammonium ion can also be adsorbed by plants roots. The sum of these N forms is described as TN [41]. The presence of plants in CWs allows a greater removal of N [42, 43].

In the first cycle, the COD removal was higher for the CWs without plants (NC*), and the CWs with the treatment levels N3 and N4 were the ones with the lowest COD removals compared to the two controls (Table 10).

**Table 10:** Average concentrations and efficiency removal of pollutants from the synthetic municipal wastewater effluent and Dunnet’s test for comparison of averages of controls with doses of GA_3_.

**Treatment**	**1st Cycle**					
	**COD**	**TN**	**TP**	**K**	**Na**	**TDS**
**g m**^**-3**^ **(%)**						
**Influent**	478	56.00	9.30	21.50	24.90	224.70
**N1**	96 (80)ª	5.22 (91)^a^	1.60 (83)^a^	3.65 (83)^a^	20.29 (19)ª	205.75 (8)ª
**N2**	78 (84)^ab^	6.72 (88)^a^	1.42 (85)^a^	3.92 (82)^a^	20.00 (20)^a^	212.48 (5)ª
**N3**	104 (78)	4.10 (93)^a^	0.90 (90)	3.36 (84)^a^	19.65 (21)	208.52 (7)ª
**N4**	115 (76)	5.29 (91)^a^	1.58 (83)^a^	4.83 (78)^a^	21.16 (15)ª	234.59 (0)ª
**NC**	83 (83)ª	5.88 (90)^a^	2.02 (78)^a^	4.27 (80)^a^	21.57 (13)ª	221.96 (1)ª
NC^*^	70 (85)^b^	36.80 (34)^b^	9.39 (0)^b^	20.72 (4)^b^	24.74 (1)^b^	376.52 (0)^b^
**2nd Cycle**						
**N1**	123 (74)^a^	7.07 (87)^a^	3.19 (66)^a^	5.22 (76)^a^	22.35 (10)ª	219.76 (2)ª
**N2**	77 (84)^b^	5.80 (90)^a^	2.60 (72)^a^	4.41 (79)^a^	21.87 (12)ª	214.49 (5)ª
**N3**	90 (81)^ab^	4.99 (91)^a^	2.71 (71)^a^	5.32 (75)^a^	21.79 (12)ª	199.48(10)ª
**N4**	114 (76)^a^	6.81 (88)^a^	2.88 (69)^a^	5.62 (74)ª	22.56 (9)ª	223.13 (0)ª
**NC**	112 (77)ª	6.31 (89)^a^	2.65 (72)^a^	5.40 (75)ª	21.36 (14)ª	210.31 (6)ª
NC^*^	66 (86)^b^	40.73 (27)^b^	10.91 (0)^b^	23.59 (0)^b^	27.48 (0)^b^	398.93 (0)^b^

For each cycle, treatment averages followed by letters ^(a)^ and/or ^(b)^ did not differ from NC and/or NC* controls, respectively by the Dunnett’s test at the 5% level of significance. COD—chemical oxygen demand; TN—total nitrogen; TP—total phosphorus; K—potassium; Na—sodium; TDS—total dissolved solids. NC-0, N1-5, N2-25, N3-50 and N4-100 μM GA_3_; NC*—control without plants in CWs.

The treatments N3 and N4 were those that obtained higher plant height and DMY in comparison with NC (1st cycle) (Table 10). As a result, it was possible to deduce that these treatments presented a greater growth of the roots. The application of GA_3_ can stimulate root growth [12]. Together with the greater development of the aerial part, these treatments may have released more exudates through the root system, resulting in higher concentrations of COD in the effluents. Exudates are secretions that release ions, free oxygen, water, enzymes, mucilage and a diversity of primary and secondary metabolites with carbon in their composition [44].

In the second cycle, the NC* treatment presented a lower COD concentration when compared to N1 and N4. The treatments that received gibberellin (5, 50 and 100 μM GA_3_) did not differentiate from the NC control. This lower COD efficiency removal in treatments with the presence of Tifton 85 bermudagrass may be related to the release of root exudates and plant senescence.

For the nutrients, Na, and TDS all treatments were superior to NC*, demonstrating the importance of plant species in CWs.

The treatment with 50 μM of GA_3_ showed a greater removal of TP and Na from the SMW compared to NC and NC* controls by the Dunnet’s test. This fact is interesting because P is one of the main nutrients responsible for eutrophication of rivers and lakes and this nutrient must be removed to comply with the legislation of disposal of effluents in water bodies. Although the efficiency of removal of TP is greater than 90% in N3, it does not comply with the legislation of Minas Gerais [45] that ranges from 0.020 to 0.025 mg L^-1^, being necessary to associate with another treatment system or recirculate therein system.

The total efficiency of CWs was obtained through the system-substrate-plant-micro-organism interaction and solar radiation. In this study, the reason for the high efficiency of nutrient removal by plants was due to the low load applied, even though this load was sufficiently great to verify the effect of GA_3_ on nutrient removal by plants, thus meeting the initial objective of the work.

The efficiency of municipal wastewater treatment of subsurface vertical flow constructed wetland cultivated with vetiver grass, observed a removal of TN of 93.9% and for TP of 90.5% for application rates of 0.8 and 0.3 g m^-2^ d^-1^, respectively [46]. These efficiencies and application rates were similar to the ones in the present study.

The TN uptake rate by the Tifton 85 bermudagrass treated with 50 μM of GA_3_ was 0.69 g m^-2^ d^-1^ of TN, as a function of an application rate of 1.02 g m^-2^ d^-1^ of TN through SMW (Table 11).

**Table 11:** Revision of application rates and total nitrogen uptake rates by Tifton 85 bermudagrass in CWs.

References	Influent	Application rate	Uptake rate	Length of Cultivation
		g m^-2^ d^-1^		d
Present study^1^	SMW	1.02	0.69	41
Matos (2008)^2^	DW	0.82	0.46	90
Fia (2011)^3^	SW	25.70	0.42	120
Matos (2010)^4^	DW	1.90	0.40	90
Jesus (2016)^5^	MW	9.20	0.79	60

^1^ Present study: average N3 data (50 μM GA_3_)

^2^ treating dairy wastewater (DW) [34]

^3^ treating swine wastewater (SW) [47]

^4^ treating dairy wastewater (DW) [9]

^5^ treating municipal wastewater (MW) [48].

The TN removal in the present study was higher than the values observed by Matos (2008), Fia (2011) and Matos (2010) [9, 34, 47]. These authors found removals of 0.46, 0.42 and 0.40 g m^-2^ d^-1^ TN from the aerial parts of Tifton 85 bermudagrass, respectively. Only Fia (2011) worked with a high rate of TN application, around 25.70 g m^-2^ d^-1^ TN, via swine wastewater [47].

Jesus (2016) observed removals of 0.79 g m^-2^ d^-1^ when treating municipal wastewater with TN application rates equal to 9.20 g m^-2^ d^-1^ in CWs with Tifton 85 bermudagrass. These values of application and removal rates were higher than the ones in the present study [48].

For different application rates and different influents (Table 12), the TP uptake rate of 0.11 g m^-2^ d^-1^ by the Tifton 85 bermudagrass treated with 50 μM of GA_3_ was higher than that observed by Matos (2008), Fia (2011), Matos (2010) and Jesus (2016). These authors verified removal rates of 0.05, 0.08, 0.04 and 0.04 g m^-2^ d^-1^ of TP of aerial parts of Tifton 85 bermudagrass, respectively [9, 34, 47, 48].

**Table 12:** Review of application rates and total phosphorus uptake rates by Tifton 85 bermudagrass in CWs.

References	Influent	Application rate	Uptake rate	Length of Cultivation
		g m^-2^ d^-1^		d
Present study^1^	SMW	0.20	0.11	41
Matos (2008)^2^	DW	0.21	0.05	90
Fia (2011)^3^	SW	7.80	0.08	120
Matos (2010a)^4^	DW	0.46	0.04	90
Jesus (2016)^5^	MW	1.30	0.04	60

^1^Present study: average N3 data (50 μM GA_3_)

^2^ treating dairy wastewater (DW) [34]

^3^ treating swine wastewater (SW) [47]

^4^ treating dairy wastewater (DW) [9]

^5^ treating municipal wastewater (MW) [48].

Although the use of GA_3_ showed potential for nutrient removal via biomass of Tifton 85 bermudagrass cultivated in CWs in the treatment of SMW, it would be interesting to conduct further field testes with higher load applications.

An application of GA_3_ in spring and another in autumn resulted in the increased dry mass of azevem and white clover [49]. Thus, responses to GA_3_ strongly depend on the time of year in which the plants were treated. And for perennial crops such as Tifton 85 bermudagrass, GA_3_ may be interacting with a fixed strategy for seasonal plant growth [50].

## Conclusions

The gibberellic acid promoted higher growth in height of Tifton 85 bermudagrass in constructed wetland systems.

The application of 50 μM of GA_3_ provided higher dry matter yield and nitrogen removal by Tifton 85 bermudagrass in constructed wetland systems for the first crop cycle.

The application of 50 μM GA_3_ was the best dose for the global removal of P and Na from synthetic municipal wastewater in the first crop cycle of Tifton 85 bermudagrass in constructed wetland systems.

The gibberellic acid applied in the second crop cycle had no effect on dry matter yield and nutrient removal by Tifton 85 bermudagrass grass in constructed wetland systems.

## Supporting information

S1 Table Dataset. Results of giberelic acid addition to batch constructed wetlands systems.
(XLSX)

## References

[1] United States Environmental Protection Agency. Guidelines for Water Reuse. Washington: Office of Water; 2012 Sect. 643p.

[2] Matos AT, Abrahão SS, Pereira OG. Desempenho agronômico de capim napier (*Pennisetum purpureum*) cultivado em sistemas alagados construídos. Eng na Agric. 2011; 19: 469–477.

[3] Çakir R, Gidirislioglu A, Çebi U. A study on the effects of different hydraulic loading rates (HLR) on pollutant removal efficiency of subsurface horizontal-flow constructed wetlands used for treatment of domestic wastewaters. J Environ Manage. 2015;164: 121–128. 10.1016/j.jenvman.2015.08.037 26363259

[4] Shutes RBE. Artificial wetlands and water quality improvement. Environ Int. 2001; 26: 441–447. 11392764 10.1016/s0160-4120(01)00025-3

[5] Kadlec RH, Wallace S. Treatment wetlands. 2nd ed. Boca Raton: CRC press; 2008.

[6] Choudhary AK, Kumar S, Sharma C. Removal of chlorinated resin and fatty acids from paper mill wastewater through constructed wetland. World Acad Sci Eng Technol. 2010; 80: 67–71.

[7] Zurita F, De Anda J, Belmont MA. Treatment of domestic wastewater and production of commercial flowers in vertical and horizontal subsurface-flow constructed wetlands. Ecol Eng. 2009; 35: 861–869.

[8] Matos AT, Abrahão SS, Lo Monaco PAV, Sarmento AP, Matos MP. Capacidade extratora de plantas em sistemas alagados utilizados no tratamento de águas residuárias de laticínios. Rev Bras Eng Agríc Ambient. 2010; 14: 1311–1317.

[9] Matos AT, Freitas WS, Martinez MA, Tótola MR, Azevedo AA. Tifton grass yield on constructed wetland used for swine wastewater treatment. Rev Bras Eng Agríc Ambient. 2010; 14: 510–516.

[10] Dunne EJ, Reddy KR. Phosphorus biogeochemistry of wetlands in agricultural watersheds In: Dunne EJ, Reddy KR, Carton OT, editors. Nutrient management in agricultural watersheds: a wetlands solution. Wageningen: Wageningen Academic Publishers; 2005 pp 105–119.

[11] Silva Coelho Y, Oliveira AAR, Caldas RC. Efeitos do ácido giberélico (AG_3_) no crescimento de porta-enxertos para citros. Pesqui Agropec Bras. 1983; 18: 1229–1232.

[12] Taiz L, Zeiger E. Fisiologia vegetal. 5th ed. Porto Alegre: Ed Artmed; 2013.

[13] Nopens I, Capalozza C, Vanrolleghem PA. Stability analysis of a synthetic municipal wastewater. Gent (Belgium): Department of Applied Mathematics, Biometrics and Process Control, 2001 7.

[14] Von Sperling M. Introdução à qualidade das águas e ao tratamento de esgotos. 4th ed. Belo Horizonte: Editora UFMG; 2013.

[15] Santos SR, Soares AA, Kondo MK, Araújo ED, Cecon PR. Crescimento e produçâo do algodoeiro fertirrigado com água residuária sanitária no semiárido de minas gerais. Braz J Irrig Draina. 2016; 21: 40–57.

[16] Matos AT, Matos MP. Disposição de águas residuárias no solo. 1st ed. Viçosa: Editora UFV; 2017.

[17] Kiehl EJ. Fertilizantes orgânicos. 1st ed. Piracicaba: Editora Agronômica "Ceres"; 1985.

[18] Silva FC. Manual de análises químicas de solos, plantas e fertilizantes. 2nd ed. Brasília: Embrapa Informação Tecnológica; 2009.

[19] Sarmento AP, Borges AC, Matos AT. Evaluation of vertical-flow constructed wetlands for swine wastewater treatment. Water Air Soil Pollut. 2012; 223: 1065–1071.

[20] Tuttolomondo T, Leto C, La Bella S, Leone R, Virga G, Licata M. Water balance and pollutant removal efficiency when considering evapotranspiration in a pilot-scale horizontal subsurface flow constructed wetland in Western Sicily (Italy). Ecol Eng. 2016; 87: 295–304.

[21] American Public Health Association, American Water Works Association, Water Environmental Federation. Standard methods for examination of water and wastewater. Washington: United Book Press; 2012; 1496 p.

[22] Hansson L, Brönmark C, Anders Nilsson P, Åbjörnsson K. Conflicting demands on wetland ecosystem services: nutrient retention, biodiversity or both? Freshw Biol. 2005; 50: 705–714.

[23] Cruz CD. Genes: a software package for analysis in experimental statistics and quantitative genetics. Acta Sci Agron. 2013; 35: 271–276.

[24] Hameed M, Ashraf M, Naz N, Al-Qurainy F. Anatomical adaptations of *Cynodon dactylon* (L.) Pers. from the Salt Range Pakistan to salinity stress. I. Root and stem anatomy. Pakistan J Bot. 2010; 42: 279–289.

[25] Atkinson NJ, Urwin PE. The interaction of plant biotic and abiotic stresses: from genes to the field. J Exp Bot. 2012; 63: 3523–3543. 10.1093/jxb/ers100 22467407

[26] Martins RG, De Camargo EC, Roberto P, Araujo DK, Silva JM, Martins MBG. Fontes e doses de giberelina no desempenho de arroz anão em biotestes. Comun Sci. 2012; 3: 306–309.

[27] Pires EJP, Maia JDG. Uso de reguladores vegetais na videira Niágara In: Maia JDG, Camargo UA, editors. O cultivo da videira Niágara no Brasil. Brasília: Embrapa, 2012 pp 282–283.

[28] Parsons AJ, Rasmussen S, Liu Q, Xue H, Ball C, Shaw C. Plant growth–resource or strategy limited: insights from responses to gibberellin. Grass Forage Sci. 2013; 68: 577–588.

[29] Zang Y-X, Chun I-J, Zhang L-L, Hong S-B, Zheng W-W, Xu K. Effect of gibberellic acid application on plant growth attributes, return bloom, and fruit quality of rabbiteye blueberry. Sci Hortic. 2016; 200: 13–18.

[30] Kaur S, Gupta AK, Kaur N. Gibberellin A_3_ reverses the effect of salt stress in chickpea (*Cicer arietinum* L.) seedlings by enhancing amylase activity and mobilization of starch in cotyledons. Plant Growth Regul. 1998; 26: 85–90.

[31] Tuna AL, Kaya C, Dikilitas M, Higgs D. The combined effects of gibberellic acid and salinity on some antioxidant enzyme activities, plant growth parameters and nutritional status in maize plants. Environ Exp Bot. 2008; 62: 1–9.

[32] Ribeiro DM, Araújo WL, Fernie AR, Schippers J, Mueller-Roeber B. Action of gibberellins on growth and metabolism of Arabidopsis thaliana plants associated with high concentration of carbon dioxide. Plant Physiol. 2012; 160: 1781–1794. 10.1104/pp.112.204842 23090585 PMC3510110

[33] Liang Y, Zhu H, Bañuelos G, Yan B, Shutes B, Cheng X, et al Removal of nutrients in saline wastewater using constructed wetlands: Plant species, influent loads and salinity levels as influencing factors. Chemosphere. 2017; 187: 52–61. 10.1016/j.chemosphere.2017.08.087 28837857

[34] Matos AT, Abrahão SS, Pereira OG. Desempenho agronômico de capim Tifton 85 (*Cynodon* spp) cultivado em sistemas alagados construídos utilizados no tratamento de água residuária de laticínios. Ambi Água. 2008; 3: 43–53.

[35] Khan NA, Mir R, Khan M, Javid S. Effects of gibberellic acid spray on nitrogen yield efficiency of mustard grown with different nitrogen levels. Plant Growth Regul. 2002; 38: 243–247.

[36] Barros AL. Morfo-anatomia e teor de nutrientes em três espécies vegetais e cultivadas em sistemas “wetland construído”. D.Sc. Dissertations, Universidade Federal de Viçosa. 2005. Available from: http://www.locus.ufv.br/handle/123456789/8841

[37] Yu K, Böhme F, Rinklebe J, Neue H-U, DeLaune RD. Major biogeochemical processes in soils-A microcosm incubation from reducing to oxidizing conditions. Soil Sci Soc Am J. 2007; 71: 1406–1417.

[38] Yü T. Physical chemistry of paddy soils. 1st ed. London: Springer; 1985.

[39] Tchobanoglus G, Burton F, Stensel HD, editors. Wastewater Engineering: Treatment and reuse. New York: American Water Works Association; 2003.

[40] Ribeiro AC, Guimaraes PTG, Alvarez VVH, editors. Recomendações para o uso de corretivos e fertilizantes em Minas Gerais: 5ª Aproximação. 1st ed. Viçosa: Sociedade Brasileira de Ciência do Solo; 1999.

[41] Matos AT. Manual de análise de resíduos sólidos e águas residuárias. 1st ed. Viçosa: Editora UFV; 2015.

[42] Herouvim E, Akratos CS, Tekerlekopoulou A, Vayenas D V. Treatment of olive mill wastewater in pilot-scale vertical flow constructed wetlands. Ecol Eng. 2011; 37: 931–939.

[43] Leverenz HL, Haunschild K, Hopes G, Tchobanoglous G, Darby JL. Anoxic treatment wetlands for denitrification. Ecol Eng. 2010; 36: 1544–1551.

[44] Bertin C, Yang X, Weston LA. The role of root exudates and allelochemicals in the rhizosphere. Plant Soil. 2003; 256: 67–83.

[45] Code of State Regulations: Deliberação Normativa Conjunta, COPAM/CERH n° 01, May (2008).

[46] Ucker FE, Almeida RA, Kemerich PDC. Remoção de nitrogênio e fósforo do esgoto sanitário em um sistema de alagados construídos utilizando o capim vetiver. Ambi Água. 2012; 7: 87–98.

[47] Fia FRL, Matos AT, Fia R, Lambert TF, Matos MP. Remoção de nutrientes por *Typha latifolia* e *Cynodon* spp. cultivadas em sistemas alagados construídos. Ambi Água. 2011; 6: 77–89.

[48] Jesus FLF. Desempenho e influência dos capins Tifton 85 (Cynodon sp.) E vetiver (*Chrysopogon zizanioides*) no tratamento de esgoto sanitário em sistemas alagados construídos. M.Sc. Thesis, Universidade Federal de Viçosa; 2016. Available from: http://www.locus.ufv.br/handle/123456789/8214

[49] Van Rossum MH, Bryant RH, Edwards GR. Response of simple grass-white clover and multi-species pastures to gibberellic acid or nitrogenfertilizerr in autumn. Proceed New Zealand Grass Assoc. 2013; 75: 145–150.

[50] Whitehead D, Edwards GR. Assessment of the application of gibberellins to increase productivity and reduce nitrous oxide emissions in grazed grassland. Agric Ecosyst Environ. 2015; 207: 40–50.

